# From Weaving Healthy Families to Weaving Healthy Communities: An Indigenist Dissemination and Implementation Approach Integrating Talking Circle Feedback

**DOI:** 10.21203/rs.3.rs-7511409/v1

**Published:** 2025-09-17

**Authors:** Catherine O’Connor, Kya Locklear, Kristi Ka’apu

**Affiliations:** Tulane University; Tulane University; Tulane University

**Keywords:** talking circle, evidence-informed practice, clinical programs, dissemination and implementation

## Abstract

A gap exists both in culturally grounded, evidence-informed Native American and Alaska Native (NA/AN) programs and in dissemination and implementation (D&I) approaches to replicate, translate, and adapt such clinical programs. D&I approaches grounded in Indigenous knowledge are needed to translate evidence-informed programs effectively into real-world settings. The purpose of this article is to (a) outline the Weaving Healthy Communities (WHC) D&I approach, with a focus on talking/sharing circles, and (b) provide results of talking/sharing circle feedback from the culturally adapted and efficacious Chukka Auchaffi’ Natana (in Choctaw) or Weaving Healthy Families program (WHF). WHF promotes resilience, wellness, and health equity, while preventing problem alcohol and other drug use, violence, and mental health conditions among NA/ANs. Using a community-based, critical ethnography with a southeastern tribe in the United States, we conducted quarterly talking circles after each cohort of WHF participants (i.e., families and facilitators) completed their active intervention. Team-based thematic qualitative data analyses were completed by the principal investigator and research team engaged in prior work with the tribe. Thematic data analysis revealed important themes of core WHF components, along with participants’ desires for extended and deepening programs that could benefit NA communities. The most frequently reported theme was talking circle benefits, coded 228 times. Within this theme, prominent subthemes included: *building trust; providing stress relief and offsetting personal burdens; learning from listening; fostering positive communication; sharing within the same peer groups; and help with problem and solution identification.*

## Introduction

A gap exists both in culturally grounded evidence-informed Native American and Alaska Native (NA/AN) programs and the dissemination and implementation (D&I) approaches to replicate, translate, and adapt such programs. This gap impedes progress to integrate efficacious programs where they are most needed. Despite culturally grounded, evidence-informed practice for NA/ANs being essential, a great need for an expansion in such services exists. Indeed, according to the Urban Indian Health Institute (2014), only 20% of problem alcohol and other drug use programs offered culturally tailored services. Not only are efficacious, culturally grounded programs for NA/ANs needed, but most tested programs, even if efficacious, are suspended after grant funding ceases. Indeed, 40% of programs end with grant funding (Blue Bird Jernigan et al., 2020). As such, efficacious programs are not being used where they are needed the most. The problem is due to gaps in (a) culturally grounded evidence-informed NA/AN programs and (b) dissemination and implementation (D&I) approaches to replicate, translate, and adapt clinical practice to new settings.

Therefore, concurrent D&I approaches grounded in Indigenous knowledge (IK) are needed to translate evidence-informed programs rapidly and effectively into real-world settings. D&I science focuses on the identification of barriers and facilitators to a program’s adoption, adaptation, integration, scaling up, and sustaining interventions. The purpose of this article is, therefore, twofold. First, we outline the Weaving Healthy Communities (WHC) D&I approach, with a particular focus on the talking/sharing circle approach to focus groups and gathering feedback. Second, we provide results of our talking/sharing circle feedback on our work on the culturally adapted and efficacious Chukka Auchaffi’ Natana (in Choctaw) or Weaving Healthy Families program (WHF). WHF promotes resilience, wellness, and health equity, while preventing problem alcohol and other drug use, violence, and mental health conditions among NA/ANs. The focus now turns to a brief description of the WHF program before providing background on talking circles as a D&I approach and intervention centering IK.

**Figure 1:**
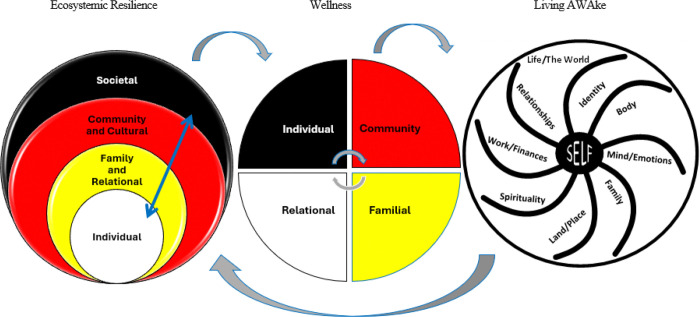
Extended Framework of Historical Oppression, Resilience, and Transcendence (FHORT) *Note.* Within the FHORT, ecosystemic or ecological risk and protective factors interact holistically andrelationally across societal, cultural and community, family and relational, and individual levels to predictkey outcomes of wellness and resilience. Wellness on the individual level includes mental, spiritual,physical, and psychological dimensions. Wellness and resilience are buffers that enable transcendencefor personal and collective wisdom that informs systemic, structural, and ecological risk and protectivefactors, giving rise to collective liberation from the colonial mindset. Copyright 2023 by C. E. McKinley.Reprinted with permission.

The WHF program is a nine-session, family-based curriculum approached with the relational, holistic, strengths-based, and Indigenist framework of historical oppression, resilience, and transcendence that was developed with the principal investigator (Burnette & Figley, 2017) and focal communities (See Figure 1). Informed by the Celebrating Families evidence-based intervention, the program was developed over a decade of community-based participatory research (CBPR; Burnette et al., 2014; McKinley et al., 2019, McKinley, Saltzman, & Theall, 2023). It has since been tested in a pilot (McKinley, Lilly, et al., 2023; McKinley, Saltzman, & Theall, 2023; McKinley & Theall, 2021) and full clinical trial. Due to space limitations, the focus of this article is on feedback about the talking circle component of the program, rather than the program itself, of which a description can be found elsewhere (e.g., McKinley, Lilly, et al., 2023; McKinley, Saltzman, & Theall, 2023; McKinley & Theall, 2021). Unlike extant comparable programs, which are school and individual based, the WHF program is family, culturally, and community based. Given families are primary socializers and have been recommended for clinical interventions (Allen et al., 2022), whole families come into WHF programming. The structure of these 2.5-hour sessions is for families to share NA/AN healthy meals, which integrates NA/AN foodways; next, they break off and receive developmentally tailored content among peer groups (e.g., parents, adolescents, 8–11 year-olds, 5–7 year-olds) where they start with talking circles for sharing; finally, families come back together a to join in experiential activities that reinforce session content. As part of our WHC D&I approach, the program is enhanced with SMS text messages after each session, reinforcing core content for a year past initial programming. Participants also receive text messages for reminders to complete and participate in program components.

## Talking Circles as an Intervention, Process, and Approach to Clinical Practice

Although, to our knowledge, talking circles have not been integrated into D&I approaches, talking circles have historically been credited for use in parliamentary procedure among the Woodland tribes of the Midwest (Running Wolf & Rickard, 2003). Representing the sacred circle, relationality, and the interconnectedness of all things (e.g., seen and unseen, earth, moon, sun, animals, plants, people), people can express thoughts in groups, honoring the perspective of the speaker, where consensus on a decision was historically reached (Avalos, 2022; Running Wolf & Rickard, 2003). Considered safe spaces to build and sometimes heal relationships, talking circles enable people to connect spiritually, intellectually, socially, and emotionally (Brown & Di Lallo, 2020). Historically, tribes have used the talking circle for learning, teaching, and listening from early childhood to adult education (Baldwin et al., 2021; Running Wolf & Rickard, 2003). Talking circles encourage instilling respect for the speaker; listening from the heart; and passing on knowledge, emotional expression, mutual support, values, and culture (Running Wolf & Rickard, 2003; Trout et al., 2018). The structures of talking circles may vary, but often the facilitator passes a small object of significance, such as a talking stick, feather, stone, crystal, or other object (Running Wolf & Rickard, 2003). The person object speaks from the heart and is not interrupted until they pass.

Talking circles have been used not only as an egalitarian, effective, and prosocial way of relating; they have also been found to be effective and healing as an intervention (Baldwin et al., 2021), not to mention a sign of political sovereignty and cultural renewal (Avalos, 2022). Indeed, talking circles have been found to have empowered community activism, increased cultural awareness, and catalyzed political mobilization among NA/ANs exposed to chronic disasters (Picou, 2000). A form of decolonizing education, pedagogical talking circles have been integrated into Canada’s Truth and Reconciliation commission, providing spaces for students to participate in relational and reciprocal learning (Barkaskas & Gladwin, 2021; Di Lallo et al., 2021). Destabilizing linear Eurocentric norms, talking circles enable open dialogue on difficult topics in a safe space (Barkaskas & Gladwin, 2021; Mehl-Madrona & Mainguy, 2014). Speakers may choose to pass if they wish, with no pressure to speak.

Although, to our knowledge, they have not been integrated into any structured D&I approach, talking circles are considered a culturally responsive evaluation process (Brown & Di Lallo, 2020). Talking circles are a replicable and ethical evaluation method to share power, empower stakeholder voice, problem solve, and increase community participation in programs’ development, implementation, and assessment. As low cost, nonhierarchical spaces for deep listening, healing, and mutual support with effect sizes comparable to treatments, talking circles are recommended as promising community-based approaches to address substance use and mental health concerns (Mehl-Madrona & Mainguy, 2014). Talking circle interventions have been found to be effective in problem substance use interventions and prevention programs among NA/AN adults and youth (e.g., Baldwin et al., 2021; Dennis & Momper, 2012; Kelley et al., 2023; Lowe et al., 2012, 2024; Momper et al., 2011; Wimbish-Tompkins et al., 2024) and have integrated storytelling, relationships, and tribal values (Baldwin et al., 2021). They have also been found to improve anger management, listening, and empathic skills among adolescent girls (Schumacher, 2014). For our program, we integrated talking circles at the beginning of peer sessions as a form of centering, to foster relational dialogue, and to foster connections across parents and children among their peer age groups. We also used them as an evaluative tool among all age groups that participated and among facilitators.

## The Weaving Healthy Families Dissemination and Implementation Approach

**Figure 2:**
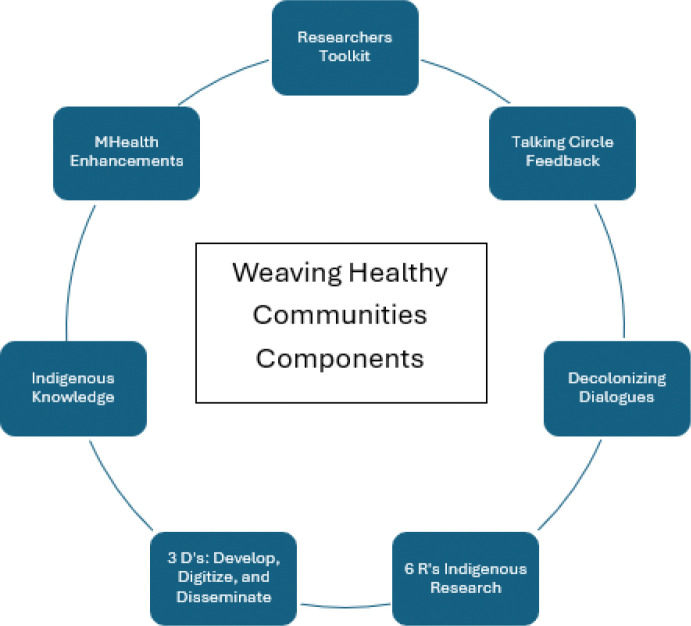
Weaving Healthy Communities (WHC) D & I Approach Components *Note*. The WHC approach integrates IK, the Researchers Toolkit (Burnette et al., 2014), the 6 R’s of Indigenous Research (Tsosie et al., 2020), decolonizing dialogues, talking circle feedback, mHealth enhancements, and introduces the three D’s of dissemination research, according to this model: develop, digitize, and disseminate.

The WHC D&I introduces three D’s of dissemination research (See Figure 2): develop, digitize, and disseminate; its approach integrates IK and CBPR with the talking circle component of the WHF curriculum in place of focus groups to gather feedback throughout and following the programming. The WHC approach centers IK in its process and approach. Though the focus of this article is the talking circle component, other key components of this D&I approach include the integration of NA/AN values identified in preliminary research, along with the six R’s of Indigenous research (Tsosie et al., 2022), including *relationship* with community, land, then environment, along with past and present generations; relevance and respect for peoples’ worldviews, ways of knowing and living, and beliefs and preferences; *responsibility* and *representation* for reciprocal and accountable relationships for stakeholders involved; and finally, *reciprocity*, a sharing and exchange based on mutual respect, trust, and honor. We integrated these values across leadership, practice, and in relationship with each other.

Other components of the WHC approach include the *Researcher’s Toolkit* (Burnette et al., 2014) for ethical and culturally sensitive approaches to work with Indigenous peoples (Burnette et al., 2014; McKinley et al., 2019, 2023b), SMS and mobile health (mhealth) enhancements for recruitment and retention of materials, leadership development and incremental living AWAke (living in alignment with agility; McKinley, 2023b, 2024; O’Connor, 2025) workshops, a structural approach to self-care and to sustainable, decolonized personal and personnel growth, development, and retention. Our approach includes community building, copublication, and leadership development with community partners (e.g., McKinley, Lilly, et al., 2023), in addition to employment of tribal personnel for the all components of the program, including food, transportation, childcare, and facilitation.

Along with strong support for the efficiency of the WHF program, our Indigenist WHC approach enabled our successful recruitment and retention during the COVID-19 pandemic that surpassed our original goals. Indeed, we recruited and retained 121 families and over 570 individual participants, while training almost 60 community-health representatives who facilitated and trained others to implement the intervention and developing an innovative talking circle focus group method to decolonize the feedback process. In addition to talking circles, we held decolonizing dialogues with the research team to ensure our approach and content did not inadvertently replicate colonial harms of historical oppression (Burnette & Figley, 2017). We held virtual decolonizing dialogues to take time as leaders to analyze our approach and process, and monthly mentor meetings for professional development.

## Methods

### Research Design

As a supplemental component of a larger critical ethnography to test the WHF program in this study, we used a community-based, critical ethnography with a southeastern tribe in the United States (Carspecken, 1996). Critical ethnography seeks to understand oppression, asymmetrical power relations, and inequities with culturally relevant and community-grounded research strategies. The talking circles replaced focus groups and were held quarterly, after each cohort of WHF participants (i.e., families and facilitators) completed their active intervention. Talking circle protocol followed the WHF talking circle protocol, developed in the curriculum. They started with general check-ins on wellness, then turned to specific feedback on the WHF program. Parallel to the WHF curriculum, talking circles were held with peer groups.

### Setting and Sample

All research procedures were approved by the tribe and the university’s institutional review board before data collection. Family participants included at least one tribal head of household female and families, including children aged 5 and above. Facilitators had experience and background working with families and completed the full WHF training program. The setting is a federally recognized tribe of the Southeast that has been blinded for the purposes of publication. The total population this sample was drawn from included 120 families and almost 600 participants (374 youth and 212 adults), along with almost 60 facilitators (*n* = 58). For background, Table 1 provides the sampling demographics of the family population, ages 12 and older in the supplemental materials. Participants recruited from our full clinical trial of participants who completed the WHF curriculum and facilitators were invited to participate in talking circles to provide feedback and received $50 renumeration on a Clincard, which could be used for cash or as debit card for participation.

### Data Collection

Talking circles were held at community facilities throughout tribal lands. From this group, in total, there were five parental talking circles with an average of 11 participants per circle and 57 participants total (*n* = 45 female; *n* = 12 male, each circle lasting an average of 48 minutes); three adolescent groups (aged 12–17) with an average of nine to 10 participants and 29 participants total (*n* = 13 female; *n* = 6 male, each lasting an average of approximately 34 minutes); and three 8–11 year-old circles with 25 total participants an average of eight to nine participants per circle, (*n* = 15 female; *n* = 10 male, each lasting approximately 16 minutes; three 5–7 year-old talking circles with 15 participants (*n* = 7 female; *n* = 8 male, each lasting approximately 15 minutes); and six facilitator talking circles, with 97 participants (*n* = 85 female; *n* = 12 male, each lasting approximately 2 hours [118.5 minutes]). Participants were able to participate in more than one talking circle; hence, the number of facilitators exceeded the total trained.

Talking circles followed a script opened with opening questions including:

- What is on your heart today; what you would like to share?
- Thinking about the four parts of the medicine wheel (i.e., mental/emotional, physical, social/familial, spiritual), where are you at today?
- Are there certain parts that need care/nurturing/healing?
- In what ways are you balanced/imbalanced?
- What do you think about the talking circle?
- How does the talking circle differ from other ways of communication or sharing information?
- What do you like about the Weaving Healthy Families program?
- Is there anything that could improve the program?
- How have you been affected by the program so far?
- What parts of the program do you think help families the most?

### Data Analysis

Team-based thematic qualitative data analysis was completed by the principal investigator and research team who had engaged in prior work with the tribe (Guest & MacQueen, 2008). Talking circles were professionally transcribed and analyzed in NVivo, a qualitative data analysis software. Immersion in the data (i.e., listening to and reading interviews multiple times) and checking for correctness were completed prior to conducting thematic analysis. The team, which included three Indigenous and one non-Indigenous person, developed hierarchical coding themes and subthemes by coding line by line, and final themes were reached by consensus.

## Results

For the WHF intervention, the talking circle was a component we integrated into each session following the family meal, after participants broke off into their respective developmental age groups (i.e., parents, adolescents, children, and younger children). As participants went to their age groups, facilitators had set up altars with materials to symbolize four elements, including water (cup of water), air (smoke from herbs), fire (burning herbs), and earth (sage, sweetgrass, or other herbs used to “smudge” at the beginning of the talking circle). This replaced the centering activity in the unadapted curriculum and was used to cleanse the space and participants prior to sharing. Participants were provided with a background and how all aspects of the talking circle were voluntary and thought to be complementary with other spiritual or faith traditions. The facilitator(s) opened with group singing, drumming, praying, or a combination thereof. Before beginning, the person with the talking object (e.g., feather, talking stick, stone, or other sacred object) spoke from the heart on the talking circle prompts that were posted for participants. Participants passed the talking object clockwise, unless the local traditions indicated otherwise, which was always prioritized over general guidelines. The group guidelines for the talking circle that were discussed and posted indicated the importance of group confidentiality, speaking from the heart, not interrupting, only one person speaking at a time, and listening with openness to the speaker.

Thematic analysis of the data revealed important themes of the core components of the WHF program, along with participants’ desires for an extended and deepening program that could benefit NA communities. The most frequently reported theme was the benefits of the talking circle, which was coded 228 times (*n* = 228). Within this theme, the most prominent subthemes included the following benefits of the talking circle: Building Trust was cited 26 times (*n* = 26); Providing Stress Relief, Healing, and Offsetting Personal Burdens (*n* = 33); Learning From Listening (*n* = 23); and Fostering Positive Communication (*n* = 24).

Though not part of the unadapted program, participants overwhelmingly spoke about the talking circle as a favorite part of the WHF program. One participant stated, “I think that’s what helps families.”

Another participant said, “The talking circle is so like healing and special.” Overall, participants thought highly of the talking circle, citing it as a safe space of support where what was said would remain confidential. Many participants had not been exposed to the talking circle and appreciated this exposure. For example, one participant said:

Talking circle is new for everyone if you weren’t born into it, but it’s not really new to our people. We’re reintroducing something that kept us alive.. .. You’re supposed to let it out. You’re supposed to talk about it to them.

Building trust (*n* = 26) was a key theme participants indicated the talking circle promoted. One participant said, “I have a trust in everybody.” Confidentiality mattered, with another participant stating, “Whatever is said here, it stays here.” A participant added:

Nobody talks about it. .. but yet, when we came into the circle. .. we had a voice, and we spoke about it.. .. When we left the circle, we didn’t go in public and, “Hey, you remember you said?” [laughing].. . .We were grown about it. We were mature about it, [be]cause we understood.

Another participant expressed comfort, saying, “I feel right at home.” This space of comfort allowed people to become vulnerable and share their experiences with each other. As one participant stated, “You just unravel yourself.” Yet, another participant indicated this safe space was instrumental in being able to share personal information with peers, stating, “I disclose information that may be very personal, but I’m okay with that.. .. I’m not a person that likes to talk.. . . That’s what I used to say, but here I’m comfortable.. .. I feel right at home.” Sharing and caring were instrumental in healing, according to a participant who stated:

At the end of the day, we all need care. We all need someone to care. The nurturing and healing, everybody needs that no matter what you got going on. Good, bad, or ugly, you still need someone to care, to nurture and to heal you.

Providing stress relief, healing, and offsetting personal burdens (*n* = 33) was another instrumental role of the talking circle. One participant indicated the immense relief provided from sharing in a group, stating, “It was like we were. .. carrying the weight of the world on us until we came to talking circle, and boom [the weight] was lifted.” Participants remarked upon these strengths and indicated the benefits of being “able to just work through it together.” A participant said, “It helps me see myself.” Another participant said it helped them realize they were “not alone,” a sentiment reiterated by another participant who stated, “I thought I was the only one.” Still another participant elaborated on the talking circle benefits, saying, “I really like to hear your stories because it helps me see myself.. . . We’re struggling through the same things, maybe at different points in our lives, but we all struggle.” Relatedly, another participant added about this theme, “to let them know that you’re not alone. .. there’s resources out there. There’s a support system that can help.” Still another person gleaned insight that the talking circle enabled healing and connection, stating, “It was a time for us to heal and connect and. .. in the process, heal somebody else.. .. There’s nothing in this world that we can’t give if we’re all together.”

Learning from listening (*n* = 23) was frequently mentioned by participants. This listening seemed to enable some transformation among some participants, as one stated, “[Friend or family] said, ‘What’s wrong with you? You’re different’ because. .. my mouth is different, the way I act is different now, because I listen. I’ve been listening since I’ve been here.” Listening was fostered by having only one person speaking at a time (i.e., person with the talking object; in our case, we used a feather), and no one interrupting. They stated, “If you have the feather, you speak, and nobody else says anything.” This practice of listening to learn was traditional for Indigenous peoples, as one remembered, “Sitting here, I remembered on weekends. .. when my mom used to go visit her aunt and grandma and cousin.” The benefits of learning from listening and continuing to learn through a storytelling approach centering Indigenous knowledge were apparent.

Fostering positive communication (*n* = 24) was indicated as a way to heal oneself and each other, relationally. One participant said, “First, you heal yourself.. .. As they say, ‘How can you help someone else if you are not focusing on yourself some?”‘ Another participant stated, “We all come from different households, but. .. [another participant] might have an answer I don’t have.. .. It’s kind of like a bridge where we all connect.” Another participant expanded upon the importance of this relational, reparative communication, stating:

We’re on the res–this is our life. This is our world. And, sometimes, the people that suffer the most, after they heal, they’re able to look up, smile, and share–not give, but share.. .. It starts with the adults, then. .. it falls down to the kids, and the kids do the same thing. It’ll get better.. .. We’re fighting a[n] uphill battle and we’re gonna [sic] make it. Everybody’s just gotta [sic] see the positive side. Everybody has something to give, something to share, something to push somebody else up.

Finally, a participant indicated the talking circle fostered balance, stating:

Balance. That’s what’s important to me, and on my heart. Because if you don’t balance things out in your life. .. one area is going to be heavier than the other.. .. It has helped us communicate better.. .. Over time, it’s become a part of us now.

## Discussion

Findings from this study highlight the centrality of talking circles as a culturally grounded approach that promotes trust, connection, and collective healing. Participants overwhelmingly identified the talking circle as the most valued component, with benefits cited 228 times across groups. Subthemes, including building trust, providing stress relief and healing, learning through listening, and fostering positive communication, reflect the multifaceted impact of this approach. As noted by participants, talking circles and their components of traditional storytelling and ceremony encouraged cultural strength and resurgence. These findings align with prior research demonstrating the efficacy of talking circles in promoting emotional literacy, relational healing, and community cohesion (Baldwin et al., 2021; Brown & Di Lallo, 2020; Mehl-Madrona & Mainguy, 2014; Running Wolf & Rickard, 2003).

Participants characterized the circle as a safe and sacred space that fostered vulnerability, mutual respect, deep listening, and relational dynamics, which contrast hierarchical, Western intervention models. Its design, grounded in voluntary sharing, the use of symbolic objects, and spiritual principles, created conditions for decolonizing dialogue and collective healing, with participants reporting developing trust, reducing stress, and engaging in open, honest communication. These outcomes align with Indigenous traditions of relational accountability and mutual support (Brown & Di Lallo, 2020; Running Wolf & Rickard, 2003) and reinforce previous findings that talking circles uphold confidentiality and support relational repair (Baldwin et al., 2021).

The WHC D&I approach demonstrates that integrating IK into program evaluation and adaptation is both feasible and essential for promoting health equity among NA/AN communities. These findings have significant implications for clinical social work practice and education. Talking circles can be integrated into therapeutic, educational, and community settings to foster cultural safety and relational healing. Core elements include arranging a nonhierarchical circle, using a culturally significant object for turn taking, and establishing agreements emphasizing confidentiality and respect. Opening and closing rituals, such as smudging, drumming, or prayer, should be adapted in collaboration with cultural advisors to reflect local traditions. Embedding these practices into curricula, agency trainings, and family or group interventions can enhance cultural responsiveness and strengthen engagement with NA/AN families. More broadly, the explored D&I approaches and principles of talking circles emphasize the value of traditional, local knowledge and collective healing processes that can be applied across diverse marginalized communities.

Reflective of WHC methods and participant feedback, some guidelines were identified for implementing efficacious healing and D&I strategies for NA/AN families:

1. Consider replacing focus groups with talking circles (ensuring cultural liaisons are represented in the process): Talking circles are a culturally congruent method for gathering feedback, fostering trust, and promoting healing. Ensure facilitators are trained in Indigenous protocols and guided by local traditions.
2. Integrate spiritual and cultural elements: Begin sessions with smudging, drumming, or prayer, and use symbolic objects (e.g., feathers, stones) to guide sharing. These elements honor Indigenous epistemologies and promote holistic engagement.
3. Center relationality and confidentiality: Establish clear group guidelines that emphasize confidentiality, respect, and not interrupting. These principles are essential for building trust and enabling participants to share openly.
4. Use developmentally tailored peer groups: Structure sessions by age group (e.g., parents, adolescents, children) to foster peer-based learning and support. Begin each group with a talking circle to center participants and promote relational dialogue.
5. Extend support beyond sessions: Follow up via text message or some other form of communication.
6. Employ tribal personnel and coleadership: Engage community members in all aspects of program delivery to ensure cultural relevance and sustainability.
7. Conduct decolonizing dialogues: Hold regular team meetings to reflect on program content and process, ensuring interventions do not replicate colonial harms.

From a D&I perspective, talking circles offer more than therapeutic benefits; they serve as an effective mechanism for gathering feedback and adapting programs across contexts. Incorporating this approach helps address persistent challenges in sustaining culturally grounded interventions, as many programs have been discontinued once funding has ended (Urban Indian Health Institute, 2014). Embedding talking circles into D&I strategies supports relational accountability, promotes community-driven adaptation, and advances long-term sustainability and self-determined problem solving for NA/AN communities. As clinical social work moves toward inclusive, decolonized models of care, the talking circle approach offers a powerful example of how Indigenous traditions can inform and transform contemporary practice.

## Limitations and Future Research

This study illustrates the role of talking circles as both a therapeutic component and an implementation strategy grounded in IK, with strengths including the use of CBPR, engagement of multiple stakeholder groups, and integration of cultural protocols. However, several limitations should be noted. Findings were drawn from a single southeastern tribe, which may limit transferability to other NA/AN communities with distinct cultural practices. Potential self-selection bias may have occurred, as participants willing to engage in talking circles could hold more favorable views of the program. The absence of long-term follow-up data restricts understanding of sustained impacts on family functioning and wellness. Additionally, youth participation posed challenges; younger children may have struggled to articulate feedback, highlighting the need for age-appropriate methods such as visual storytelling or participatory arts.

Future research should assess the scalability of talking circles across diverse tribal contexts and evaluate culturally specific adaptations. Combining quantitative measures with qualitative insights would strengthen the evidence base and support broader dissemination. Further investigation is needed into how facilitator characteristics shape participant experiences and the feasibility of virtual or hybrid models to improve access in rural or remote settings. Finally, developing youth-centered and technology-enhanced engagement strategies will be essential for extending the reach and cultural relevance of Indigenist implementation approaches.

## Figures and Tables

**Table 1:** Participant Demographics

Demographic characteristics	Adult sample (*n* = 218)	Adolescent sample (*n* = 202)

	*n* (%)	*n* (%)

Participant sex		

Male	61 (28.0)	87 (43.0)

Female	157 (72.0)	115 (57.0)

Race/ethnicity (*n* = 221[check all apply])		
Native American/American Indian	214 (98.2)	354 (100.0)
White	3 (1.4)	2 (0.6)
Black or African American	3 (1.4)	5 (1.4)
Hispanic/Latino	1 (0.5)	6 (1.7)
Language(s) spoken (*n* = 218)		
Native/Tribal language	141 (64.7)	78 (31.3)
English	172 (78.8)	163 (65.5)
French/Spanish/other	7 (3.2)	8 (3.9)

Relationship status (*n* = 199)		

Married	60 (30.2)	-

Single	79 (39.7)	-

Cohabiting	40 (20.1)	-

Divorced/engaged/widowed	20 (10.0)	-

Annual household income (*n* = 199)		

$0–$15,000	65 (32.6)	-
$15,001–$25,000	40 (20.1)	-

$25,001–$50,000	50 (25.1)	-
> $50,001	44 (22.1)	-

Financial difficulty (*n* = 199)		

Extremely difficult or very difficult	44 (22.1)	-

Somewhat difficult	52 (26.1)	-

A little difficult	63 (31.7)	-

Not at all difficult	40 (20.1)	-

Employment status (*n* = 186)		
Working full time	127 (63.8)	-
Other	59 (29.6)	-

Education (*n* = 199)		

Less than high school	27 (13.6)	-

High school/GED	58 (29.1)	-

Some college	58 (29.1)	-

Vocational/technical or associates	29 (31.1)	-

Bachelor’s/master’s/other degree	27 (24.6)	-

Household Type (*n* = 233 [check all that apply])		
Single parent	67 (28.7)	67 (38.7)
Two-parent	129 (55.3)	75 (43.4)
Blended or extended	37 (15.8)	31 (17.9)

	*M (SD)*	

Average age at pretest	35 (range 18–73)	13.80 (range 12–17)

Average household size at pretest	5.0 (range 2–14)	-

Average number of biological children	3.42 (3.52)	-

*Note.* Reported from the adult and youth sample at pretest only.

## Data Availability

Data must be requested to the Principal Investigator and required approval from associated tribe(s).
